# The Chronological Trigger: The Orchestra Between Homeobox Genes and the Circadian Clock During Development

**DOI:** 10.1111/boc.70027

**Published:** 2025-07-25

**Authors:** Joice de Faria Poloni, Bruno César Feltes

**Affiliations:** ^1^ Department of Biophysics Federal University of Rio Grande do Sul Porto Alegre Rio Grande do Sul Brazil; ^2^ Children's Cancer Institute Porto Alegre Rio Grande do Sul Brazil

**Keywords:** circadian rhythm, development, evolution, homeobox, homeodomain

## Abstract

As master regulators of embryonic development, regulating homeobox genes is fundamental for developmental biology. Despite the growth of multiple topics regarding fine‐tuning homeobox gene expression, the discussion on how the circadian rhythm affects their control and vice‐versa still needs to be improved. Due to the intrinsic importance of the circadian clock and its impact on several molecular mechanisms, including development and pregnancy, the interplay between this mechanism and homeobox genes becomes a meaningful discussion. This work aims to review and critically discuss the crosstalk between homeobox genes and circadian regulation in multiple organisms, focusing on differentiation and developmental mechanisms. A considerable focus is given to new perspectives on the topic.

## Graphical abstract

Homeobox genes associated with the circadian rhythm and the development of specific tissues, playing crucial roles in regulating cellular differentiation and the formation of structures during embryonic development.

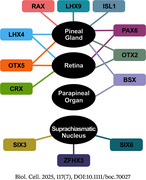

## 1 Introduction

Eukaryotic development is a broad topic, connected by dozens of environmental and molecular factors that orchestrate the genesis and growth of an organism. Discussing such a topic inevitably leads to a multitude of variables that, to some extent, could influence embryogenesis. Among these variables, it is undeniable that the homeobox genes play a crucial role during development. Homeobox comprises a superfamily of genes characterized by an evolutionarily conserved helix‐loop‐helix‐turn‐helix domain named homeodomain. Homeodomain‐containing proteins play an essential part as transcription factors (Holland 2013; Bürglin 2011). Since the activation of homeobox during development triggers the regulation of several targets (Soshnikova 2014; Divoux et al. 2017), they require strict molecular control mechanisms to fine‐tune their expression, such as epigenetic repression (Deschamps and Duboule 2017; Montavon and Duboule 2013), control by miRNA (Divoux et al. 2017; Gregory et al. 2008; Godfrey et al. 2018; Fantini et al. 2018), and long noncoding RNA (Dong et al. 2018; Su et al. 2017; Zheng et al. 2018). Homeobox genes are intrinsically related to various molecular pathways besides development—DNA repair (Feltes 2019), different neurological functions (Philippidou and Dasen 2013; Waite and Martin 2015), several diseases (Joo et al. 2016; Rodrigues et al. 2016; Soto et al. 2016; Tomioka et al. 2014; Bäckström et al. 2017), and metabolism (Zhou et al. 2018; Wang et al. 2017, 2018), to name a few. Hence, studying the mechanisms that regulate their expression is an ever‐expanding subject.

Nevertheless, one of the often‐overlooked aspects of homeobox genes during development is their interaction with the circadian rhythm. Circadian rhythm is an intrinsic and autonomous biological feature of all living organisms to adapt and prepare their cellular functions in response to external modifications, such as light and temperature (Chaix et al. 2016). As a primal biological feature, the circadian rhythm is related to several biological functions and diseases, like Alzheimer's Disease (Homolak et al. 2018), cancer (Nirvani et al. 2017; Maiese 2017), allergies (Paganelli et al. 2018), sleep disorders (Kim and Duffy 2018), and pregnancy (Pears et al. 2018); it is even known to influence the intestinal microbiome (Voigt et al. 2016) and the immune system (Curtis et al. 2014). Hence, it is unsurprising that a whole branch of the pharmaceutical industry is exclusively dedicated to researching and designing drugs to regulate the circadian rhythm (Ozturk et al. 2017; Tahara and Shibata 2014; Winter and Soehnlein 2018). More importantly, in our context, the circadian machinery has already been observed to impact development (Vallone et al. 2007; Seron‐Ferre et al. 2007). Consequently, this crosstalk between the circadian molecular machinery and homeobox gene expression presents an interesting and curious case from a developmental perspective.

This work reviews the relationship of different homeobox genes with the circadian rhythm and circadian‐associated proteins during the developmental process. All molecular aspects, either direct or indirect, will be considered as long as they play a role in the circadian regulation and development. We will also outline core concepts crucial to understanding the relationship between homeobox genes and the circadian rhythm; however, please note that the focus of this review is not a detailed description of these topics, and the reader will be redirected to specific reviews. New perspectives and critical evaluations are conducted throughout the review.

## 2 Assembling the Clockwork Machinery

### 2.1 The Wings of Time: The Core Circadian Regulators

The molecular circadian regulation revolves around a 24‐h autoregulatory feedback loop that impacts the transcription and translation of several targets, named clock‐controlled genes (CCG) (Gerhart‐Hines and Lazar 2015; Partch et al. 2014). This endogenous timing mechanism coordinates an organism's behavior and physiology related to the day‐night cycle. In this pathway, a core regulatory mechanism controls bioenergetic metabolism, allowing for adjustments in physiological responses to external stimuli. CCG and functional clock machinery are expressed in most cell types; however, mammals have the suprachiasmatic nucleus (SCN) in the hypothalamus that serves as a clock‐dedicated structure, while in birds, reptiles, amphibia, and fish, the pineal gland is the one that plays a role in the coordination of the circadian oscillation and the synchronization of the clock through cycling systemic signals (Foulkes et al. 2016).

**FIGURE 1:**
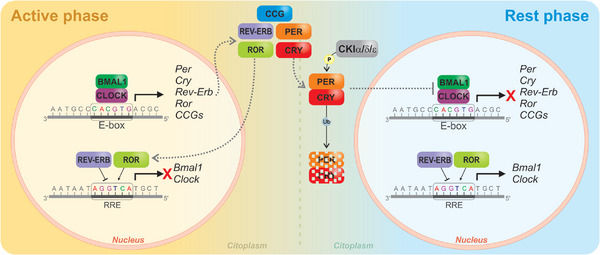
The core circadian clock mechanism. In the main loop, transcriptional‐translational feedback loop, the CLOCK/BMAL1 heterodimer binds to E‐box elements within the promoters of target genes during the active phase, promoting the expression of Per, *Cry*, *Ror*, *Rev‐erb*, and numerous clock‐controlled genes (CCGs) via binding to E‐box elements within the promoters. Following the accumulation of PER and CRY, the PER/CRY complex translocates to the nucleus during the rest phase, where it inhibits CLOCK/BMAL1 activity, thereby suppressing further transcription of their own genes and other CCGs. As PER and CRY proteins accumulate in the cytoplasm, they form heterodimers and undergo phosphorylation by CKIα, CKIδ, and CKIε, which regulates PER turnover and modulates their stability and targets them for ubiquitination and proteasomal degradation. In a secondary loop, REV‐ERBα/β and RORα/β/γ compete for binding to ROR‐response elements (RREs) in *Bmal1* promoters, where REV‐ERBs act as repressors and RORs as activators, thus finely tuning *Bmal1* expression across the circadian cycle. Regulating their expression levels by regulating BMAL1 transcription. Except for the E‐box and consensus sequence of RRE, all other sequences are merely illustrative.

In mammals, the central subject of this review, this core mechanism begins with the heterodimerization of the proteins BMAL1 (ARNTL) and CLOCK, which not only activates the transcription of CCG but also binds to the E‐box enhancer of the proteins PER1‐2 and CRY1‐2. Following their transcription and translation in the cytoplasm, PER and CRY proteins accumulate and form a stable heterodimeric complex (Partch et al. 2014). This PER/CRY heterodimer then actively translocates back into the nucleus (Takahashi 2017). Once in the nucleus, the PER/CRY complex directly interacts with the CLOCK‐BMAL1 heterodimer, binding to it and sterically hindering its ability to bind to E‐box elements on DNA and recruit co‐activators (Takahashi 2017). This direct interaction leads to the transcriptional repression of Per and *Cry* genes, along with other CCGs, effectively creating the negative autoregulatory feedback loop (Takahashi 2017). The subsequent degradation of PER and CRY proteins, primarily mediated by phosphorylation by Casein Kinase I alpha (CKIα), CKI delta (CKIδ), and CKI epsilon (CKIε) kinases, marks them for ubiquitin‐dependent proteasomal degradation (Fagiani et al. 2022; Hirota et al. 2010). The reduction of PER and CRY levels alleviates the inhibition on CLOCK‐BMAL1, thereby triggering the restart of the cycle. Concurrently, in a crucial secondary regulatory loop, the CLOCK‐BMAL1 heterodimer also promotes the expression of the nuclear receptors REV‐ERBα/β and RORα/β/γ (Partch et al. 2014; Takahashi 2017; Fagiani et al. 2022). These proteins then compete for binding to ROR‐response elements (RREs) located in the *Bmal1* promoter. Specifically, REV‐ERBα/β acts as transcriptional repressors of *Bmal1*, while RORα/β/γ function as transcriptional activators of *Bmal1* (Partch et al. 2014). This competitive binding at RREs provides a critical mechanism for fine‐tuning BMAL1 expression levels, thereby modulating the amplitude and stability of the overall circadian molecular response (Partch et al. 2014; Takahashi 2017). This intricate interplay of positive and negative feedback loops, along with accessory factors, ensures robust and precise circadian rhythms. Other proteins have been described to play a role in this mechanism, including ubiquitin complexes, transcription factors, kinases, phosphatases, epigenetic‐related proteins, and post‐transcriptional regulators, among others (Gerhart‐Hines and Lazar 2015; Partch et al. 2014; Chong et al. 2012; Ben‐Shlomo and Kyriacou 2002; Kojima et al. 2011). Figure 1 shows a summary of this core machinery.

Aside from BMAL1, CLOCK, PER, and CRY, another target to be discussed as a circadian regulator is the Timeless protein (TIM). TIM has a well‐described circadian function in *Drosophila melanogaster*, but it is usually left out of mammals' canonical, or even the essential, clockwork machinery (Bloch et al. 2013). When discussing the circadian machinery, it is undeniable that the fruit fly *D. melanogaster* holds a special place in the history of the timekeeping mechanism—most genes related to the circadian clock were first identified in *D. melanogaster* (Rosato et al. 2006). All master regulators discussed previously, such as period (per), clock (clk, clkJrk in flies), timeless (tim1), and cycle (cyc, an ortholog of BMAL1), among many others, are present in *D. melanogaster* and share similar functions with their mammalian counterparts (Bloch et al. 2013; Zhang and Kay 2010; Meuti and Denlinger 2013; Konopka and Benzer 1971). Beyond the identification of core clock genes, studies in *D. melanogaster* have provided fundamental insights into the cellular and circuit‐level mechanisms that govern circadian timekeeping. Research has demonstrated that the molecular clock components control neuronal membrane excitability, influencing ion channel activity to generate rhythmic neuronal firing (Flourakis et al. 2015). This principle of molecular clock‐controlled excitability is crucial for rhythmic output and is broadly conserved across species, although the specific ion channels and detailed regulatory pathways may differ (Flourakis et al. 2015). Furthermore, high‐resolution mapping of the synaptic connectome of the *Drosophila* circadian clock has revealed intricate neural circuits and specific connections between clock neurons and their downstream targets, which are essential for orchestrating rhythmic behaviors and sleep‐wake cycles (Reinhard et al. 2024). Although mammalian brain circuits are more complex for connectome mapping, the fundamental principle of networked clock neurons coordinating physiological rhythms is conserved (Reinhard et al. 2024; Donlea et al. 2014).

TIM, especially, plays a varied role in DNA repair, embryonic development, carcinogenesis, and neurological disorders (Mazzoccoli et al. 2016). Mammalian TIM has already been demonstrated to possess closer homology to its fly counterpart (Sangoram et al. 1998) and is part of the negative feedback loop by regulating CLOCK‐BMAL1 activity through the modulation of PER expression (Sangoram et al. 1998; Barnes 2003). Despite this fact, the mammalian TIM is not considered a core circadian modulator, and even in the two previously cited articles, the different authors diverge about TIM's evolutionary role. Nevertheless, it is worth mentioning TIM because *TIM‐1* knockdown has proved to cause embryonic lethality in *Caenorhabditis elegans* and mice (Chan et al. 2003; Gotter et al. 2000).

The main properties of the circadian clock are highly conserved across evolution, even in phylogenetically distant organisms (Foulkes et al. 2016; Stanton et al. 2022). As the core components of the circadian clock are conserved between distant species, it is suggested that the origin of this mechanism comes from a bilaterian common ancestor (Stanton et al. 2022). However, evolutionary divergence has led to species‐specific adaptations in the molecular circadian clock, with divergences in their expression patterns and regulation. This has led to both conserved elements and unique features of circadian regulation, reflecting each species’ evolutionary history and ecological niche (Stanton et al. 2022; Jabbur and Johnson 2022; Jabbur et al. 2024).

### 2.2 Melatonin Production and Its Impact on Both Development and Circadian Rhythm

To explain circadian regulation in mammals and its relationship to development, it is essential to briefly review the role of melatonin in influencing the circadian rhythm and its connection to development. Melatonin is a hormone produced by the pineal gland, an endocrine gland that, in humans, is located in the center of the brain (Macchi and Bruce 2004). During embryogenesis and fetal development, melatonin is predominantly supplied by maternal transfer in most mammalian species. In human fetuses, maternal melatonin is detected in circulation, with levels slowly rising from 24 weeks of gestation, peaking in the third trimester (D'Angelo et al. 2020). During the last trimester of pregnancy, the fetal biological clock is developed under entrainment of maternal circadian rhythms (D'Angelo et al. 2020). While the anatomical structures responsible for melatonin synthesis, such as the pineal gland and the SCN, are present at birth, an endogenous circadian rhythm of melatonin secretion is typically absent until approximately three months of age, resulting in low levels of endogenous melatonin in newborns infants (D'Angelo et al. 2020; Gomes et al. 2021). This transient postnatal melatonin deficiency is primarily attributed to the delayed maturation of the pineal gland itself. During postnatal development, there is a marked increase in LIM homeobox 4 (Lhx4) within the pineal gland (Paditz 2024). Lhx4 is involved in the pineal gland's differentiation and development, also contributing to the regulation of melatonin synthesis in pinealocytes (Paditz 2024). The complete development and differentiation of the pineal gland, including its pinealocytes (as the primary site of melatonin synthesis), primarily occur during the first year of life (Paditz 2024). Concurrently, the development and synaptic coupling of the associated predominantly noradrenergic neural pathways and vessels also occur within this critical first year (Paditz 2024). These resulting low levels of endogenous melatonin may be consequently associated with sleep disorders, infant colic, and increased crying in babies (Paditz 2024). Intervention studies indicate that this deficiency should be compensated for through nutrition, such as breastfeeding (Paditz 2024). Specifically focusing on postnatal development, exogenous melatonin administration has demonstrated significant neuroprotective roles against perinatal brain injuries (Pang et al. 2021; Nacarkucuk et al. 2024). These benefits include influencing neurogenesis and neuronal survival during critical windows of brain development (Pang et al. 2021).

Moreover, most eukaryotes have some level of molecular synchronism that reacts to a light‐dark (LD) cycle. In higher vertebrates, light detection occurs exclusively in the retina, which converts the stimulus of electromagnetic waves into chemical reactions encoded in the brain and regulates the circadian rhythm. The retina has its circadian clock, which is adapted to respond to fluctuations in electromagnetic stimuli over a 24‐h period. This response affects all levels of cellular adaptation, from gene expression and protein synthesis to cell survival and neurotransmitter release. Overall, all levels of electromagnetic stimulation affect the LD cycle, including day length (i.e., the duration of light exposure), light intensity, and the period of no significant light exposure (the dark cycle). These are not only influenced by geographical locations but also depend on lifestyle (e.g., environmental conditions, such as “breaks” in work and school environments, exposure to screen time, etc.) and/or existing diseases or conditions, such as cataracts, and vitamin deficiency (Wirz‐Justice et al. 2021; Dresp‐Langley 2020). For a detailed description of the retinal clock control, please refer to (Jandot et al. 2022).

For the purpose of this review, it is important to explain the LD cycle due to its close association with melatonin synthesis. Melatonin synthesis is inhibited during high‐light exposure, peaking during the dark period. However, exposure to artificial light can also partially inhibit its synthesis, such as long hours in front of computers, cell phones, and domestic light. In this sense, blue light appears as a major player in fast melatonin synthesis inhibition (Okano et al. 2017; Chellappa et al. 2011).

**FIGURE 2:**
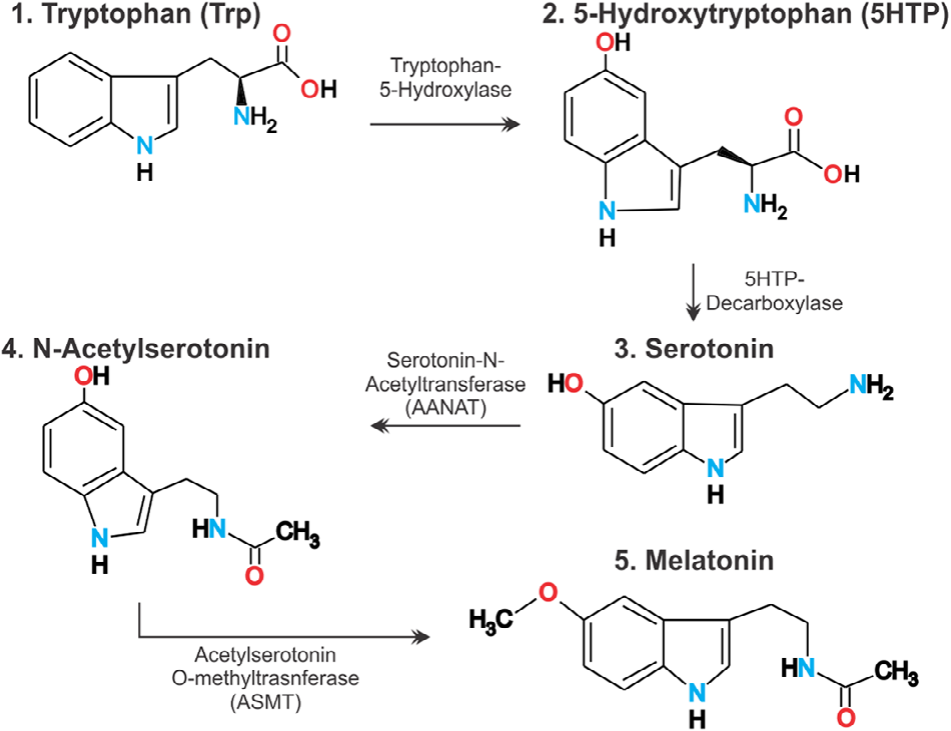
Melatonin synthesis pathway with its associated enzymes. These molecules were drawn using ChemSketch [www.acdlabs.com/products/draw_nom/draw/chemsketch].

Melatonin synthesis begins with the conversion of tryptophan to 5‐hydroxytryptophan, which is converted to serotonin, N‐acetylserotonin, and later to melatonin (Figure 2). However, enzymatic levels of aralkylamine N‐acetyltransferase (AANAT), as well as for the levels of serotonin and melatonin, are different during the day, implying a dynamical and rhythmic pathway of action that culminates in high levels of melatonin during the night (Sapède and Cau 2013). Melatonin binds to the MT1 and MT2 receptors, coupled to G proteins that control cAMP and cGMP levels by inhibiting adenylate and guanylyl cyclase activity, ultimately decreasing PKA level (Sapède and Cau 2013; de Faria Poloni et al. 2011; do Amaral and Cipolla‐Neto 2018). Overall, dark periods significantly reduce the release of serotonin, which in turn directly impacts melatonin production. The release of other neurotransmitters, such as GABA and dopamine, can also play a role in partially inhibiting melatonin synthesis (Reiter 1991; Claustrat and Leston 2015; Monteleone et al. 1997).

Melatonin is present in all animals. It was primarily described as an antioxidant that evolved, acquiring complex biological functions until it became intrinsically related to the circadian machinery and the sleep cycle in vertebrates (Schippers and Nichols 2014; Zhao et al. 2019). *Danio rerio* lacking *aanat2* showed a drastic change in sleep behavior, although not in the expression of circadian genes (Gandhi et al. 2015). Melatonin treatment also suppressed the effects of *Clock/+* mice with a functional *aanat* (Shimomura et al. 2010). Melatonin was seen to induce the expression of *per1* and *per2* (Agez et al. 2009; Kandalepas et al. 2016), and *bmal1* (Agez et al. 2007), but all those effects were through indirect means, especially by affecting PKC and Nuclear Orphan Receptors (NOR) expression and activity. In the adipose tissue of obese and chronic jet‐lagged mice, melatonin induced Clock expression and promoted cell cycle (Liu et al. 2017). In human myometrial smooth muscle cells, MT2 expression was induced by CLOCK‐BMAL1 (Beesley et al. 2015), and in rats, it was demonstrated that melatonin administrations caused the expression of *per2* and *bmal1* in the heart (Zeman et al. 2009; Zeman 2013). However, despite all the evidence showing how melatonin is crucial to the sleep cycle, or the facts showing that this hormone can induce the expression of *bmal1*, *clock*, and per, the role of melatonin in the core circadian molecular machinery remains a matter of debate (Sapède and Cau 2013; Fisher et al. 2013; Borjigin et al. 2012).

Aside from influencing circadian regulation, melatonin has already been observed to have neuroprotective roles in neurological disorders (Ramos et al. 2017), such as dementia (Alagiakrishnan 2016; Chakraborti et al. 2015), multiple sclerosis (Farez et al. 2016), Alzheimer's disease (Shukla et al. 2017; Wang and Wang 2006), and Fragile X Syndrome (Won et al. 2017). Likewise, it has a positive impact on cardiovascular function (Sun et al. 2016; Nduhirabandi and Maarman 2018), respiratory diseases (Habtemariam et al. 2016), and metabolism (Cipolla‐Neto et al. 2014). Regardless of its various roles in multiple biological backgrounds, the most pivotal aspect of melatonin, to our context, is how it was already described to modulate embryonic development, a topic that started years ago (Davis 1997; Weaver 2005).

Melatonin exhibits circadian rhythmicity after certain phases of development (Csernus et al. 2007; Kazimi and Cahill 1999; Mirmiran et al. 2003), and it has been extensively studied for its involvement in neurodifferentiation by affecting different signaling pathways and proteins (de Faria Poloni et al. 2011; Colella et al. 2016; Sinha et al. 2018; Ghareghani et al. 2017; Shu et al. 2016; Sarlak et al. 2013). In humans, melatonin passes the placenta and fetal blood barriers and influences fetal rhythmicity and development through melatonin receptors found in fetal SCN as early as 18 weeks (Van Drunen and Eckel‐Mahan 2023). Perhaps one of the most studied aspects of melatonin in development is its impact on the maturation of oocytes and embryos, as well as embryonic and fetal development (Carlomagno et al. 2018; Reiter et al. 2014), which was investigated in mice models (Zhang et al. 2017; Ishizuka et al. 2000; He et al. 2015), bovine (Pang et al. 2018), fish (Lombardo et al. 2014; Danilova et al. 2004), goats (Soto‐Heras et al. 2019), and porcine (Jin et al. 2017; Lee et al. 2018; Wang et al. 2017). It is important to note that most commonly used laboratory mouse strains, such as C57BL/6, are genetically incapable of producing melatonin. Despite this deficiency, these animals are still able to develop functional circadian rhythms and exhibit normal clock gene oscillations, suggesting that melatonin is not essential for the formation or basic operation of the circadian clock during development. Yet, even though they can still exhibit functional circadian rhythms, melatonin is still a vital molecule that bridges several circadian functions and is deeply associated with development, as will be described further.

## 3 Homeobox Genes, Development, and Their Relationship With the Circadian Modulation

It is already established that the retina possesses an endogenous circadian clock that regulates the LD cycle and is independent of the SNC (Okano et al. 2017; Felder‐Schmittbuhl et al. 2017). The pineal gland appears to have an evolutionary origin with retinal photoreceptors (Mano and Fukada 2006), which implies a robust molecular link between their functions.

One of the oldest homeobox genes of interest regarding the circadian rhythm in the eye, or photoreceptors, in general, is Crx. It was first related to photoreceptors' survival, differentiation, and function in 1997 by different researchers (Furukawa et al. 1997; Chen et al. 1997; Freund et al. 1997; Swain et al. 1997), permanently rooting it as a crucial homeobox for the retinal circadian rhythm, a finding later confirmed by subsequent studies (Tsuji et al. 2007; Møller et al. 2017). Crx was later identified as a potential target for pineal gene expression (Li et al. 1998). Several studies have investigated its role as a modulator of the pineal and retinal gene expression system. For example, Rovsing et al. showed that the pineal gland of mice lacking Crx is morphologically similar to the wild type (Rovsing et al. 2011). Crx was identified as a modulator of *Aanat* expression in rat pinealocytes (Rohde et al. 2014) and affects temperature and rhythms in mice (Rovsing et al. 2010). Crx is one of the top examples of Homeobox genes modulating the circadian rhythm through proper tissue development and melatonin synthesis.

Another homeobox gene that has been heavily discussed for its interplay with circadian regulation is the Orthodenticle homeodomain protein‐5 (Otx5), as it has been extensively reported to be essential for pineal differentiation, melatonin synthesis, and the core circadian machinery in multiple species (Vuilleumier et al. 2007; Gamse et al. 2002; Plouhinec 2003). Briefly, studies for this protein reported that Otx5 is interrelated to the transcription of *Aanat2* and *Reverbα* in *D. rerio* pineal gland (Gamse et al. 2002; Nishio et al. 2008; Appelbaum et al. 2005), as well as able to enhance the expression of the Bmal1/Clock heterodimer in mouse fibroblasts (Appelbaum et al. 2005, 2006). The same relationship between Otx5, Bmal1/Clock, and Aanat2 was observed in gilthead seabream (*Sparus aurata*) (Zilberman‐Peled et al. 2007). In the same topic, Otx2 expression levels have been detected in developing rats' pineal gland and retina (Rath et al. 2006; Nishida et al. 2003), and they were recently described as working together with Crx to control the expression of *Aanat* and *Asmt* in the pineal gland (Rohde et al. 2019). Interestingly, *Oxt2* has its expression modulated by Clock in *Xenopus laevis*, indicating another layer of complexity between the Oxt family and circadian regulation (Green et al. 2001). Although Otx5 and Otx2 are part of the eye differentiation system (Rath et al. 2006; Shen and Raymond 2004), most studies agree that they are not required for circadian regulation in this tissue.

The role of Pax6 in eye development is paramount, as Pax6‐mutants exhibit early arrest of eye development and reduced proliferation of retinal progenitors (Oron‐Karni et al. 2008). Pax6 is widely expressed in the developing brain, including the pineal gland. Pax6‐mutant failed to develop the subcommissural organ and posterior commissure, and the pineal gland (Estivill‐Torrús et al. 2001). Furthermore, Pax6 is targeted directly by Clock by a conserved E‐box consensus sequence present in its promoter in different embryonic tissues, such as the brain, eye, and pancreas (Morgan 2004). Mutant Pax6 mice showed a loss of circadian rhythm and modifications in feeding behavior (Chhabra et al. 2020).

Moreover, LIM Homeobox 9 (Lhx9) is another homeobox gene expressed in the developing mouse forebrain, where its knockout resulted in the incomplete development of the pineal gland (Yamazaki et al. 2015). In mice, its expression is essential for normal sleep behavior (Dalal et al. 2013). The *Drosophila* Lhx9 homolog Ap was related to light‐driven arousal and showed expression driven by a (Shimada et al. 2016). However, newer homeobox genes are described to play roles in pineal development and the circadian clock. Using a conditional knockout of the Retina And Anterior Neural Fold Homeobox (Rax) for the eye and the pineal gland in mice, Rhode et al. in 2017 demonstrated that the lack of Rax culminated in numerous malformations in the optic nerve and the pineal tissue (Rohde et al. 2017). The authors detected reduced levels of Aanat after adrenergic stimulation. Aanat is usually regulated by norepinephrine at night, affecting cAMP levels and, consequently, its expression (Rohde et al. 2017).

Another fresher homeobox gene seen to influence melatonin synthesis and affect the circadian rhythm is the ISL LIM Homeobox 1 (Isl1) gene. Zhang et al. in 2018 demonstrated, in pigs, that *Isl1* is expressed in the pineal gland, and its knockdown decreases *Aanat* expression levels, whereas its overexpression was able to increase it by more than 2.5 fold (Zhang et al. 2018). The authors confirmed that melatonin levels responded to changes in Isl1 expression status, where its knockdown diminished melatonin levels by 29.2%, and its overexpression increased it by 1.3‐fold. They further explored this data and found that norepinephrine stimulates Isl1 expression, and this stimulation occurs through the ERK pathway. The data gathered in this study also supports the notion that homeobox genes are commonly associated with melatonin synthesis above any other circadian modulator.

The brain‐specific homeobox (Bsx) is yet another new addition to the known targets that have been identified to influence pineal development. Schredelseker and Driever, in 2018, published a remarkably comprehensive work on the role of Bsx in pineal development. In summary, they demonstrated that Bsx expression in the pineal anlage region starts at 14 h of development, peaks at 24 h, and is co‐expressed with the tryptophan‐5‐hydroxylase 1a (tph1a) gene in melatonin‐secreting cells (Schredelseker and Driever 2018). The author's experiments concluded that *bsx* mutants displayed morphological and differentiation defects in the parapineal organ, as well as diminished expression of Otx5 and LIM homeobox 2b (Lhx2b), which suggests that these mutants may also affect the pineal gland. Loss of tph1a and aanat2 was observed in *bsx* mutants' pinealocytes, suggesting impairments in melatonin synthesis, agreeing with the well‐documented role of homeobox genes in this pathway. In agreement with *bsx* crucial role in pineal development, Carstensen et al. showed that *bsx* ^−/−^ adult zebrafish completely lacked the pineal gland. Likewise, *bsx* ^−/−^ mutants consistently displayed diminished *aanat2*, *crx*, and *otx5* mRNA levels (Carstensen et al. 2022).

*Bsx* was also observed to be expressed in melatonin‐producing pinealocytes of adult mice, peaking its expression during the dark period (Carstensen et al. 2020). Inhibition of *Bsx* by siRNA also generated higher levels of *Otx2* and *Pax4* mRNA, but no impact was observed in melatonin synthesis‐associated genes (*Tph1*, *Aanat*, and *Asmt*). Thus, the effects of Bsx slightly diverges from its role during zebrafish development, as seen in (Schredelseker and Driever 2018), where *bsx* mutants affected *tph1a* and *aanat2* levels. This aligns with the established behavior of homeobox genes, which play distinct roles in adult individuals of various species. Yet, it suggests that the molecular effects of homeobox genes in circadian regulation and melatonin synthesis should not be generalized.

## 4 The Tips of New Icebergs in Connecting Homeobox Genes and the Circadian Rhythm During Development

Consistent with the growing interest in understanding the role of homeobox genes in circadian regulation, Hertz et al. studied the role of homeobox genes in phototransduction (Hertz et al. 2021). The authors demonstrated that mRNA levels of Otx2, Crx, and Lhx4 were high in the pineal gland during the dark period in both LD and DD conditions in postnatal mice. In contrast, mRNA levels of these three genes remained constant in the retina, regardless of the light conditions. *Pax4* also displayed higher mRNA levels in L‐D transition in the pineal gland and the retina, and smaller levels in DD in the retina. Furthermore, the knockdown of Otx2, Crx, and Lhx4 by RNAi in pinealocytes showed a significant reduction of mRNA levels of multiple phototransduction‐associated genes. In this sense: (i) Grk1, Gucy2d, Slc24A1, and Sag appear as targets of all three homeobox; (ii) Guca1b was regulated by Crx and Otx2; (iii) Rcvrn, Gnat2, and Cngb1 mRNA levels were by the knockdown of Crx and Lhx4; and (iv) Pde6b was exclusively altered by Otx2 siRNA. This study revealed a profound relationship between homeobox genes and the transcriptional regulation of phototransduction genes in the retina, underscoring the significance of Otx2, Crx, and Lhx4 in controlling the circadian machinery. Although this work did not study any developmental relationships, the fact that Crx and Otx2 were already deeply associated with pineal development allows for a broader discussion of these genes and phototransduction genes during development. Likewise, the loss of *Pax6* and *Otx2* function in mice affected retinogenesis and prevented pineal gland development, whereas *Crx* exerts a regulatory role in the adult pineal gland, controlling melatonin synthesis (Nishida et al. 2003; Rohde et al. 2019, 2014; Estivill‐Torrús et al. 2001; Remez et al. 2017; Furukawa et al. 1999). In addition, Rcvrn was already associated with retina development (Sharma et al. 2003), and mutations in Pde6b are also well‐known to affect rod cell development (Han et al. 2013). However, this notion should not be extended to all homeobox genes. Otx2 and Crx were already associated with the pineal developmental process and circadian regulation during developmental stages, and Lhx4 is part of pituitary development (Sheng et al. 1997); however, in terms of its connection to circadian regulation‐associated genes, it was only observed during postnatal stages (Hertz et al. 2020). However, Lhx4 is still associated with circadian regulation because its knockout by siRNA led to lower levels of *Aanat*, *Asmt*, and *Tph1*.

Homeobox genes are also observed to influence circadian regulation, but are not associated with the molecular basis of the clock. In 2017, Wilcox et al. demonstrated the role of the Zinc finger homeobox 3 (Zfhx3) transcription factor in regulating the circadian clock in mice (Wilcox et al. 2017). By utilizing tamoxifen‐induced Cre mice, the authors demonstrated that adult homozygous Zfhx3 mutants exhibited absent Zfhx3 expression after tamoxifen treatment in the SCN. They also detected a shortening of the period in the DD condition and increased activity during the light phase and the first moments of the dark phase in the LD cycle. This notion is strengthened by a study from Hughes et al. in 2021 (Hughes et al. 2021). Eight‐week‐old mice were maintained under a 12:12 LD cycle for 1 week, followed by 2 weeks of DD and 2 weeks of LL. Separately, mice were exposed to 7–10 days of reduced LD. They showed that Zfhx3 is expressed in the retina of developing and adult mice, with protein levels decreasing over time. They also observed that mutant Zfhx3 exhibited reduced corneal thickness, although this did not affect its distribution across different retinal cells or the expression of Cry1 and Cry2 mRNA. Despite this lack of molecular response, the authors noticed a higher cortical response to light stimulus and increased activity during LL experiments. The authors concluded that Zfhx3 may play a role in areas receiving information from the retina, thereby affecting brain response and altering the animals’ behaviors. The mentioned works clearly show that Zfhx3 is associated with external responses to the LD cycle, but the lack of more in‐depth investigations about Zfhx3's association with melatonin production or other CCG leaves a fertile ground for new works.

Another interesting study performed by D'Autilia et al. in 2010 (D'Autilia et al. 2010) studied the roles of the Xenopus Bsx (Xbsx), homolog of the human BSX, in regulating pineal photoreceptors' cell cycle in *X. laevis* development. The authors revealed that Xbsx expression is inversely proportional to proliferation—higher *Xbsx* expression inhibits proliferation and promotes photoreceptor differentiation. More specifically, *X. laevis* embryos in LD conditions cyclically expressed Xbsx, with Xbsx inhibiting cell proliferation during the night. Moreover, *Xbsx* knockdown causes S‐phase entry, whereas stimulation of *Xbsx* expression causes S‐phase exit and increased differentiation. While this study did not investigate the relationship between Xbsx and the melatonin pathway, other studies focusing on different circadian‐related genes in other species may be of further interest.

Another series of studies demonstrated that the SIX Homeobox 6 (Six6) gene is regulated in a circadian manner, but it did not establish a connection to the core circadian molecular machinery or the developmental process. In this sense, Clark et al. in 2013 demonstrated that *Six6*‐null mice in DD conditions displayed aberrant circadian behavior, which the authors later correlated with impairments in the development of the SCN (Feng and Bass 2016). The same group later affirmed that Six3 and Six6 had higher expression during the day in mice pituitary and that Six6 was responsive to estradiol treatment (Xie et al. 2015). These works were not solely focused on the circadian rhythm; thus, the experiments were not in‐depth enough to fully assess the six family roles in this machinery. Nevertheless, the authors showed that Six6 influences SCN development, with some rhythmicity in adults, opening a new window of exploration of these genes in developing organisms. On the other hand, Hoffman et al. showed that Six3 positively regulates *Per2* transcription (Hoffmann et al. 2021). The authors established that Six3 and Vax1, other two homeobox genes, not only regulated Per2 expression but were also associated with circadian locomotor activity and ovulation efficacy in mice. In male mice, Six3 also enhanced the expression of Bmal1, with no effects on fertility (Meadows et al. 2022). Both works focused on the effects of Six3 in postnatal/adult stages. Thus, the effects during the early stages of development remain to be established.

Nair et al. described a role for the homeobox transcription factor, scarecrow (scro), in regulating circadian locomotor activity and development in *D. melanogaster* (Nair et al. 2020). In this sense, scro was observed to negatively regulate the expression of the pigment‐dispersing factor (pdf) neuropeptide, which is associated with controlling circadian locomotor activity. The expression of *scro* led to arrhythmic behavior under DD and failed light‐on anticipation in LD conditions. After ruling out that *scro* overexpression is not cytotoxic, the authors proved that the homeodomain of scro was essential to its binding to the promoter region of pdf, where scro homeodomain mutants lacked the negative regulation of pdf and displayed normal locomotor activity. Remarkably, scro truncated mutants proved to be lethal during fly development, with 20% of individuals dying at embryonic development and the remaining dying at the L1 or L2 larval stage. This work proved that scro has a role in the circadian locomotor activity but did not assess its role in regulating the expression of core CCG nor study the impact of scro in CCG during development, leaving an open question on how/if scro can be a new homeobox gene influencing circadian rhythm. Although other works showed that scro mRNA is not present in clock‐neurons in adult individuals (Nagoshi et al. 2010). A more in‐depth investigation of the scro relationship with the core circadian molecular machinery during development could tackle this question.

Finally, Das et al. showed that *HoxA10* was downregulated in the uterus during implantation in mice exposed to LL during pregnancy (Das et al. 2022). The authors exposed pregnant mice to LL conditions and observed arrhythmic expression of core circadian regulators in SCN, such as *Bml1*, *Clock*, *Per1*, and *Cry1*. A similar pattern was observed in the mRNA of these genes in the uterus, including *Per2*. Although no direct study was performed to observe how desynchronization between the SCN and peripheral clock systems occurs and how this relates to the expression of HoxA10, the deregulation of a HOX gene by the circadian rhythm is of utmost importance. Hox genes are master regulators of body pattern formation, regionalization, and cell differentiation during development. During pregnancy, HoxA10 and HoxA11, especially, are crucial for embryo implantation (Du and Taylor 2016). However, our understanding of the relationship between the circadian rhythm, CCG, and Hox genes is limited, making it one of the most fertile areas of discussion on this topic.

### 4.1 The Quest for an Ideal Study

Designing an experiment to study the influence of a gene on the circadian rhythm is a challenging task, as this mechanism can affect multiple metabolic and developmental pathways. This challenge increases when homeobox genes come into the scene because they genuinely control tissue formation, generating an analytical problem for all experiments in vivo and in situ that try to observe their impact on the circadian machinery. Most homeobox genes are directly connected to pineal development and function, such as Rax (Rohde et al. 2017), Otx5 (Vuilleumier et al. 2007; Gamse et al. 2002; Nishio et al. 2008; Appelbaum et al. 2005), Otx2 (Rath et al. 2006; Nishida et al. 2003; Hertz et al. 2021), Crx (Li et al. 1998; Rovsing et al. 2011; Hertz et al. 2021), and Bsx (Carstensen et al. 2022; Shi et al. 2004). When they are not just related to development, they are, at least, expressed in the pineal tissue, like Isl1 and Lhx4 (Zhang et al. 2018; Hertz et al. 2021, 2020). Thus, it is difficult to point out the real source of the effect when a study is working with knockdowns, knockouts, and/or mutations in a given homeobox gene: the observed morphological and behavioral effects are a response of a direct impact on CCG expression or a reflection of a broad tissue impairment that would affect multiple pathways anyway? Studies must consider this complexity and exercise caution when making strong observations without proper design, such as implying changes in developmental features without morphological observations or roles in molecular pathways without proper experiments to observe transcriptional activation or protein‐protein interactions. Behavioral studies could be enriched if integrated with in vitro experiments exclusively constructed to observe molecular effects—hence isolating the developmental bias. Likewise, the work cited in (Zhang et al. 2018) provides a valuable solution for investigating the impact of homeobox genes on melatonin synthesis, as it examined melatonin levels to confirm that the molecular connection affects melatonin synthesis. The work executed by Schredelseker and Driever (2018) is a great example of a thorough study that considers almost all aspects of a comprehensive investigation (e.g., morphological observations, molecular pathways involved, and relationships to the developmental process) and can serve as an example of proper protocol. Different approaches, like the ones performed in Rovsing et al. (2010), which investigated temperature rhythms, are instrumental as an additional parameter to add to behavior studies.

**FIGURE 3:**
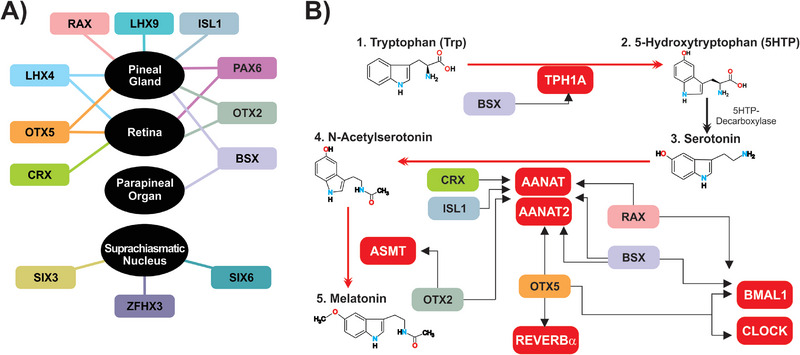
Summary of the interplay between homeobox genes and circadian regulators during development. (A) Homeobox genes and the tissues in which they were reported to play a role in their development, function, or circadian rhythm. (B) Model depicting the melatonin pathway, Bmal1 and Clock, and the homeobox genes they are associated with. Most homeobox genes are related to melatonin synthesis, affecting almost all steps in this pathway. RT, retina; PG, pineal gland; PO, parapineal organ; SCN, suprachiasmatic nucleus.

Figure 3 presents a summary of all the data discussed so far.

## 5 Additional Perspectives

### 5.1 Circadian Regulation in Sponges

Little is known about circadian regulation in sponges or its relationship to development. However, evidence points out that the siliceous spicules that compose the skeleton of sponges can harvest certain wavelengths, transmit the light within the sponge's body, and generate endogenous light through luciferase (Müller et al. 2013). Due to the role context of circadian regulation in sponges (e.g., deep‐sea location, absorption of specific wavelengths, and lack of neural system), more studies on the subject would be of valuable contributions to the knowledge surrounding not only the circadian regulation but its relationship to development since homeobox genes were already detected in sponges (Ren et al. 2018). A complete and comprehensive review of such mechanisms can be found in Müller, 2013 (Müller et al. 2013).

### 5.2 Plunging into the Dark

After so much data regarding the role of homeobox genes in retinal development and function, how could we uncover more about these processes and their connection to homeobox genes and the circadian rhythm? The answer may lie in the “missing links.” As discussed, the circadian rhythm is a primitive response to environmental changes that even non‐eukaryotic organisms possess. However, the development of optic features wholly changed how we reacted to the LD cycle. Homeobox genes appear to be strongly interconnected with the development of photosensitive organs and melatonin, indicating that they primarily emerged in regulating this process late in evolution. The question is: how are homeobox genes first related to the response to the LD cycle in the retina if they were at all before the retinal and pineal tissues first appeared in the vertebrate lineage? Studying early vertebrates with intermediate optic features might answer this question. Hagfishes, for example, possess an early form of ocular tissue, which lies between non‐forming and forming imaging eyes and lacks the pineal gland. Hence, studying these animals could shed light on how homeobox genes were first associated with the circadian rhythm. For the interested reader, Collins et al. in 2009 (Collin et al. 2009) provided an exceptional discussion on photoreceptors in the early vertebrates, which inspired this subtopic.

### 5.3 Hox, Retinoic Acid, and the Clock

Surprisingly, few studies have investigated the role of Hox genes in the development of the retina and pineal gland in the context of the circadian rhythm. Hoxc4 expression was reported to be overexpressed in mice pineal glands lacking Crx (Rovsing et al. 2011), but no studies further evaluated if this finding impacted the circadian clock. Hox genes are primarily present during embryonic phases of development, and their expression is usually absent in adults or restricted to constantly regenerating tissues, such as the endometrium (Du and Taylor 2016), which could explain the lack of studies trying to evaluate their role in the timekeeping mechanism and in the brain. However, they might act on a different but converging pathway in the circadian rhythm: the retinoic acid (RA) pathway. Studies indicate that multiple Hox genes are expressed in various brain regions, even in areas where they are not typically expressed during development, suggesting a late role for these genes beyond regional patterning (Hutlet et al. 2016; Lizen et al. 2017). In this sense, one possible action of Hox genes could be regulating RA, which is already confirmed to happen during embryogenesis through multiple mechanisms (Daftary and Taylor 2006). RA‐induced genes pathways have been extensively studied and are considered key modulators of the core circadian machinery, primarily RA receptors α and β (RARα‐β) and Retinoid X Receptors (RXRs) (Navigatore‐Fonzo et al. 2013). Future studies on the relationship between Hox genes and RA signaling could provide more information about the roles of homeobox genes and the circadian rhythm. A complete review of retinoids' influence on the brain can be found in (Ransom et al. 2014).

## 6 Conclusions

Both development and the circadian rhythm exhibit a well‐conserved molecular pathway, directly indicating their evolutionary roots. The circadian clock was established as a survival mechanism, adapting an organism to changes in its environment; thus, it is not surprising that it could severely affect the developmental process. The current scientific literature suggests that, in vertebrates, this major connection originated with the development of the pineal gland, which influences melatonin synthesis. Most of the homeobox genes discussed in this review appear transcriptionally linked to this pathway by regulating several of its key enzymes, with the notable exception of 5HTP‐decarboxylase. This is a curious phenomenon because the circadian mechanism was already conserved in invertebrates. The emergence of a more complex neurological system may represent a turning point in the evolutionary divergence of this mechanism. More studies in organisms with intermediate optic tissues, such as hagfishes, could help explain the role of homeobox genes in modulating the circadian clock before the pineal tissue evolved. Additionally, works focusing on unraveling the roles of Hox genes in the circadian clock, especially through the RA pathway, can be a new fertile ground for future perspectives.

## Conflicts of Interest

The authors declare no conflicts of interest.
